# 135. Association Between Implementation of the Centers for Medicare & Medicaid Services Sepsis Performance Measure (SEP-1) and Outcomes in U.S. Hospitals

**DOI:** 10.1093/ofid/ofab466.135

**Published:** 2021-12-04

**Authors:** Chanu Rhee, Tingting Yu, Rui Wang, Sameer S Kadri, David Fram, Huai-Chun Chen, Michael Klompas

**Affiliations:** 1 Harvard Medical School and Harvard Pilgrim Health Institute, Boston, MA; 2 Harvard Medical School and Harvard Pilgrim Health, Boston, Massachusetts; 3 National Institutes of Health Clinical Center, Bethesda, Maryland; 4 Commonwealth Informatics, Waltham, MA; 5 Harvard Medical School and Harvard Pilgrim Health Care Institute, Boston, Massachusetts

## Abstract

**Background:**

In October 2015, CMS began requiring U.S. hospitals to report compliance with the Severe Sepsis/Septic Shock Early Management Bundle (SEP-1). We evaluated the impact of SEP-1 implementation on sepsis treatment patterns and outcomes using detailed clinical data from diverse hospitals.

**Methods:**

We conducted a quasi-experimental interrupted time-series analysis of adults admitted to 114 hospitals in the Cerner HealthFacts dataset from October 2013-December 2017 with suspected sepsis (defined by blood culture orders, SIRS criteria, and acute organ dysfunction) within 24 hours of hospital arrival. The primary outcome was quarterly short-term mortality rates (in-hospital death or discharge to hospice). Secondary outcomes included lactate testing and administration of anti-MRSA or anti-Pseudomonal beta-lactam antibiotics within 24 hours of hospital arrival. Generalized estimating equations with robust sandwich variances were used to fit logistic regression models to assess for immediate SEP-1 impact and changes in quarterly trends after October 2015, adjusting for baseline characteristics and severity-of-illness.

**Results:**

The cohort included 117,510 patients with suspected sepsis on admission. Lactate testing rates increased over the study period (61.9% pre-SEP-1 vs 77.9% post-SEP-1) with a significant immediate increase in risk-adjusted testing rates after SEP-1 (OR 1.34, 95% CI 1.04-1.74) (Figure 1). There was also an increase in utilization of anti-MRSA (20.6% pre vs 23.2% post-SEP-1) and anti-Pseudomonal antibiotics (30.1% vs 39.8%), %), but these trends began before SEP-1 implementation. Unadjusted short-term mortality was similar in the pre vs post-SEP-1 periods (20.3% vs 20.4%). SEP-1 was not associated with either an immediate change (OR 0.94, 95% CI 0.68-1.29] or quarterly trend change (OR 1.00, 95% CI 0.97-1.04] in risk-adjusted short-term mortality (Figure 2).

Figure 1. Quarterly risk-adjusted rates of A) lactate testing, B) anti-MRSA antibiotic administration, and C) anti-Pseudomonal beta-lactam antibiotic administration within 24 hours of hospital presentation for patients with suspected sepsis before and after SEP-1 implementation in Q4 2015.

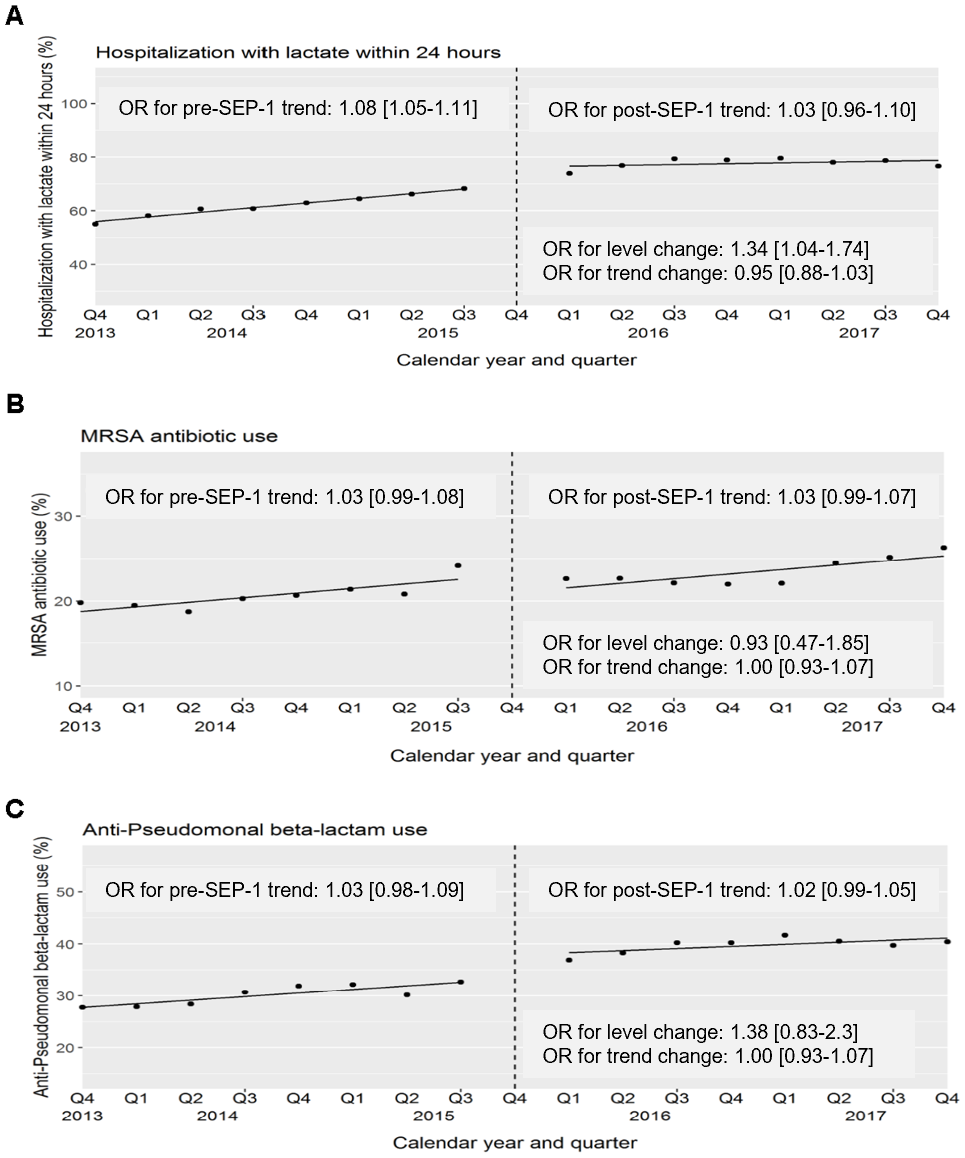

All models included time (in quarters), an indicator of the post-SEP-1 implementation period (starting Q12016, to allow for evaluation of an immediate policy effect), and a two-way interaction term to assess whether SEP-1 implementation resulted in a change in trend. When data suggested no change in trend, models were also fit without this interaction term; this yielded a significant association for an immediate level change in lactate testing (OR 1.34 [95% CI 1.04-1.74]) but not antibiotic utilization. All analyses were adjusted for patient severity of illness and baseline characteristics including age, sex, race, initial vital signs (systolic blood pressure, temperature, respiratory rate, heart rate), and initial laboratory results (creatinine, platelet count, bilirubin, white blood cell count) if done within 24 hours.

Figure 2. Quarterly risk-adjusted outcomes of patients with suspected sepsis before and after SEP-1 implementation in Q4 2015: A) In-hospital death or discharge to hospice, and B) In-hospital death alone.

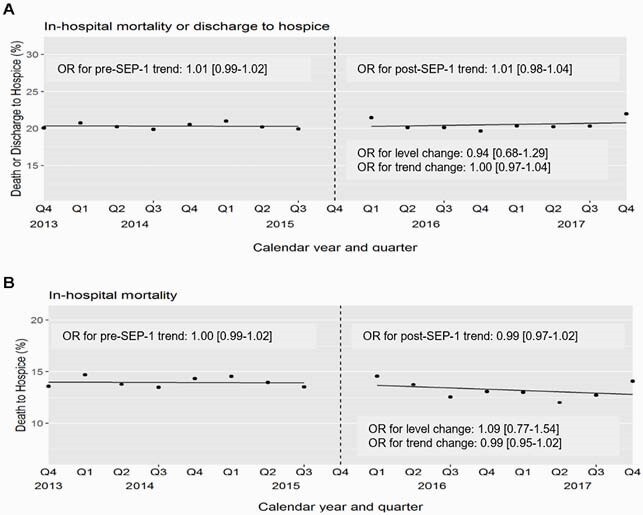

Models included time (in quarters), an indicator of the post-SEP-1 implementation period (starting Q12016, to allow for evaluation of an immediate policy effect), and a two-way interaction term to assess whether SEP-1 implementation resulted in a change in trend. Analyses were adjusted for patient severity of illness and baseline characteristics including age, sex, race, initial vital signs (systolic blood pressure, temperature, respiratory rate, heart rate), and initial laboratory results (creatinine, platelet count, bilirubin, white blood cell count) if done within 24 hours.

**Conclusion:**

SEP-1 implementation was associated with an immediate increase in lactate testing rates, no significant change in already-rising rates of broad-spectrum antibiotic use, and no change in short-term mortality rates for patients with suspected sepsis in a large cohort of hospitals. Other approaches to decrease sepsis mortality may be warranted.

**Disclosures:**

**Chanu Rhee, MD, MPH**, **UpToDate** (Other Financial or Material Support, Chapter Author) **Sameer S. Kadri, MD, MS, FIDSA**, Nothing to disclose **Michael Klompas, MD, MPH**, **UpToDate** (Other Financial or Material Support, Chapter Author)

